# Soot and Smoke

**Published:** 1908-01-04

**Authors:** Wm. Nicholson

**Affiliations:** Chief Smoke Inspector. Health Office, Sheffield.


### "SOOT AND SMOKE."

*To the Editor of* THE HOSPITAL.

SIR,—I have read with very great interest and pleasure your article on "Soot and Smoke" in the current issue of THE HOSPITAL, and what you state is only too true. I have written three books on the smoke question, given lectures before the Royal Sanitary Institute, Smoke Abatement Conferences, etc., and in December 1905 I went to India for six months *re* smoke abatement for the Government, and while there many nuisances were abated owing to Government action. Four years ago I suggested to the Local Government Board the creation of a "Smoke Department" and the appointment of inspectors to advise local authorities and compel them to administer the smoke law. I was informed "the said suggestions were placed on record for consideration in the event of Parliament deciding to take action on the smoke question"; but nothing has been done by the Government.

If the technical and daily Press, medical men, and the general public were to commence a Smoke Abatement crusade, very shortly Government would take action, and the result would quickly be an atmosphere fit to breathe, improved public health, and not only a great saving to the manufacturer but great public profit.

Yours faithfully,

WM. NICHOLSON, Chief Smoke Inspector.

Health Office, Sheffield.

December 27.

